# Hexadecenoic Fatty Acid Isomers in Human Blood Lipids and Their Relevance for the Interpretation of Lipidomic Profiles

**DOI:** 10.1371/journal.pone.0152378

**Published:** 2016-04-05

**Authors:** Anna Sansone, Evanthia Tolika, Maria Louka, Valentina Sunda, Simone Deplano, Michele Melchiorre, Dimitrios Anagnostopoulos, Chryssostomos Chatgilialoglu, Cesare Formisano, Rosa Di Micco, Maria Rosaria Faraone Mennella, Carla Ferreri

**Affiliations:** 1 ISOF, Consiglio Nazionale delle Ricerche, Bologna, Italy; 2 Lipinutragen srl, Lipidomic Laboratory, Bologna, Italy; 3 Institute of Nanoscience and Nanotechnology (INN), N.C.S.R. Demokritos, Athens, Greece; 4 University of Napoli “Federico II”, Department of Clinical Medicine and Surgery, Napoli, Italy; 5 University of Napoli “Federico II”, Department of Biology, Napoli, Italy; University of Geneva, SWITZERLAND

## Abstract

Monounsaturated fatty acids (MUFA) are emerging health biomarkers, and in particular the ratio between palmitoleic acid (9*cis*-16:1) and palmitic acid (16:0) affords the delta-9 desaturase index that is increased in obesity. Recently, other positional and geometrical MUFA isomers belonging to the hexadecenoic family (C16 MUFA) were found in circulating lipids, such as sapienic acid (6*cis*-16:1), palmitelaidic acid (9*trans*-16:1) and 6*trans*-16:1. In this work we report: i) the identification of sapienic acid as component of human erythrocyte membrane phospholipids with significant increase in morbidly obese patients (n = 50) compared with age-matched lean controls (n = 50); and ii) the first comparison of erythrocyte membrane phospholipids (PL) and plasma cholesteryl esters (CE) in morbidly obese patients highlighting that some of their fatty acid levels have opposite trends: increases of both palmitic and sapienic acids with the decrease of linoleic acid (9*cis*,12*cis*-18:2, omega-6) in red blood cell (RBC) membrane PL were reversed in plasma CE, whereas the increase of palmitoleic acid was similar in both lipid species. Consequentially, desaturase enzymatic indexes gave different results, depending on the lipid class used for the fatty acid content. The fatty acid profile of morbidly obese subjects also showed significant increases of stearic acid (C18:0) and C20 omega-6, as well as decreases of oleic acid (9*cis*-18:1) and docosahexaenoic acid (C22:6 omega-3) as compared with lean healthy controls. Trans monounsaturated and polyunsaturated fatty acids were also measured and found significantly increased in both lipid classes of morbidly obese subjects. These results highlight the C16 MUFA isomers as emerging metabolic marker provided that the assignment of the double bond position and geometry is correctly performed, thus identifying the corresponding lipidomic pathway. Since RBC membrane PL and plasma CE have different fatty acid trends, caution must also be used in the choice of lipid species for the interpretation of lipidomic profiles.

## Introduction

**Fig 1:**
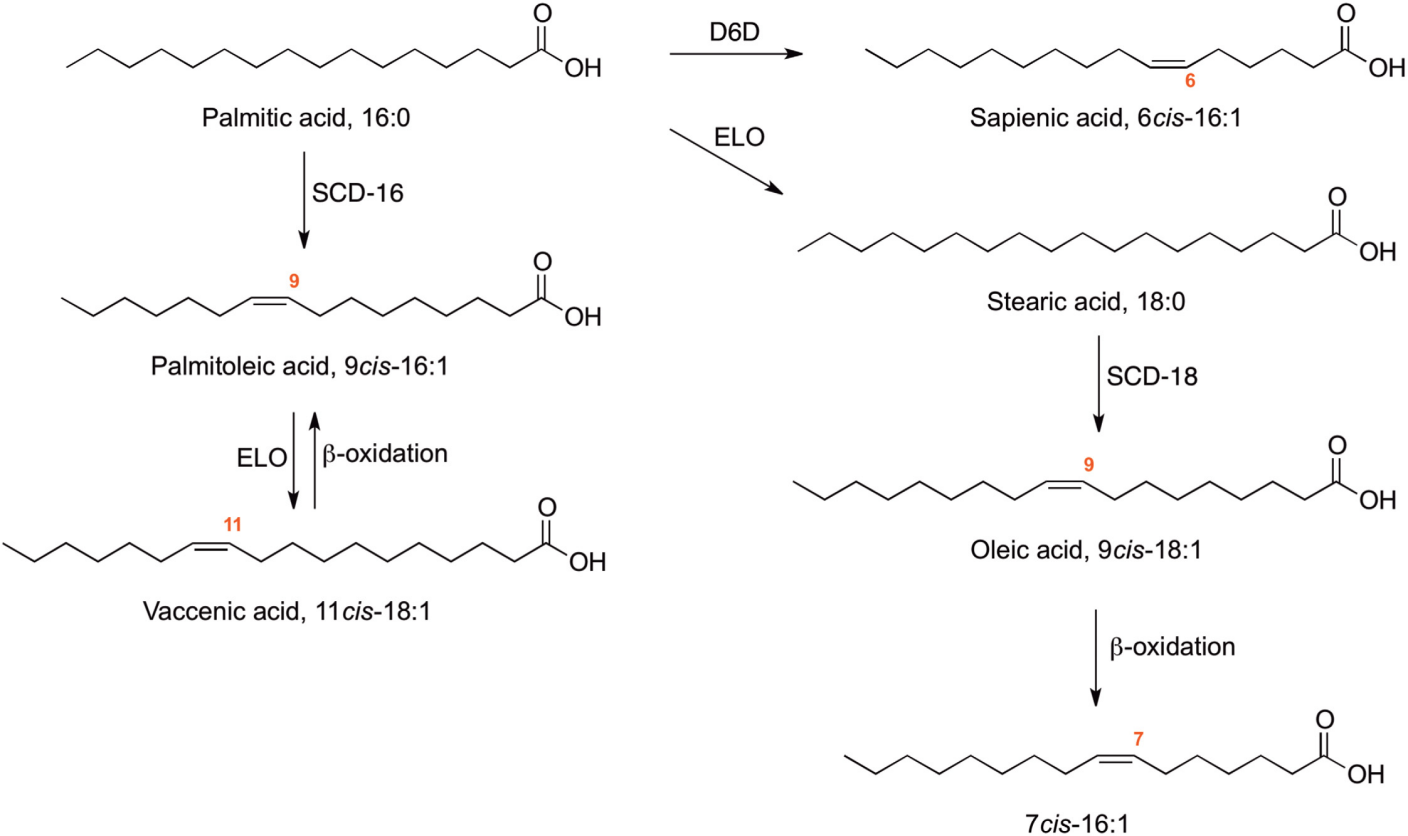
Biosynthetic pathways for the formation of three positional isomers of the hexadecenoic fatty acid family (C16 MUFA). The formation of 9*cis*-16:1 (palmitoleic acid) occurs from palmitic acid via delta-9 desaturase (SCD-16) and from vaccenic acid via beta-oxidation. The formation of 6*cis*-16:1 (sapienic acid) occurs from palmitic acid (via delta-6 desaturase, D6D) and the formation of 7*cis*-16:1 occurs from oleic acid via beta oxidation. Oleic acid is obtained from stearic acid (18:0) via delta-9 desaturase (SCD-18).

Examination of the fatty acid content in human tissues and the dynamic interpretation of their remodeling in the body contribute to the identification of lipidomic profiles, in connection with fat metabolism and nutrition in health and diseases [1–3]. In particular, human plasma is subject of intense research due mainly to the remarkable diversity of lipids and challenge of lipidomic analyses [4–8], and to the easiness of the withdrawal procedure, together with the high potential for biomarker discovery and definition of lipidomic phenotypes [9,10]. The data on overweight and obese individuals gathered so far pointed attention to the metabolic transformation of saturated fatty acid (SFA) to monounsaturated fatty acids (MUFA), with a focus on the conversion of palmitic acid to palmitoleic acid (9*cis*-16:1) by the activity of the delta-9 desaturase enzyme (stearoyl coenzyme A desaturase, SCD-16) [11–15], as shown in Fig 1. The evaluation of the SFA-MUFA pathways and calculation of fatty acid desaturase indexes are reported in diverse conditions, ranging from sportive activity, diet and pregnancy to diabetes and cancer [16–18]. In the hexadecenoic fatty acid (C16:1) family the activity of palmitoleic acid was highlighted as a lipokine, stimulating muscle insulin action as well as preventing lipogenesis and fat accumulation in the liver [19]. More recently, it was found to trigger the signalling cascade for preventing the high-fat induced pro-inflammatory macrophage polarization [20]. Within the C16:1 MUFA family sapienic acid (6*cis*-16:1) is a positional isomer with a shifted position of the double bond along the carbon atom chain, that is obtained from palmitic acid by the activity of delta-6 desaturase enzyme (D6D). This fatty acid was thought to be associated only to skin metabolism and sebum triglycerides [21]. Recently our group reported for the first time its presence as component of human lipoprotein lipids and plasma cholesteryl esters (CE) [22]. An efficient protocol of separation and quantification of C16 MUFA positional and geometrical fatty acid isomers was carried out. Consequently, the metabolic fate of palmitic acid processed by delta-6 and delta-9 desaturases could be proposed according to Fig 1. More recently, enzymatic studies have demonstrated the competition between palmitic acid and PUFA for Fatty Acid Desaturase *FADS*2 (D6D), highlighting the relevance of the sapienic acid evaluation in health and diseases [23]. On the other hand, the geometrical trans isomers of C16 MUFA (6*trans*-16:1 and 9*trans*-16:1) attract interest as marker of an endogenous isomerization process, which is known to occur under cellular stress by the attack of free radicals—in particular sulfur-centered radicals—affecting the natural cis geometry of lipids [24]. It is worth underlining that trans fatty acid isomers were reported in the plasma of obese children [25, 26] whereas trans hexadecenoic isomers are not yet correlated to any health status. As far as the hexadecenoic positional isomerism is concerned, we are also aware of another positional isomer produced by the oleic acid (9*cis*-18:1) degradation pathway, namely 7*cis*-16:1 (Fig 1).

In this work we report for the first time the comparison of the fatty acid composition of red blood cell (RBC) membrane phospholipids (PL) and plasma cholesteryl esters (CE) in morbidly obese patients (n = 50) and in healthy lean individuals (n = 50), focusing on the palmitic acid metabolism to C16 MUFA. These two lipid classes are connected through the activity of lecithin cholesterol acyl transferase (LCAT), that transfers the fatty acid chain from PL to CE, an emerging pathway involved in human pathologies [27]. The general aim of this work is to give new insights of the MUFA pathway regarding the positional and geometrical isomers, in order to enhance the understanding of lipidomic profiles in health and diseases.

## Materials and Methods

### Ethics Statement

The present study was conducted according to the guidelines laid down in the Declaration of Helsinki and all procedures involving human patients were approved from the Ethical Committee “C. Romano” for biomedical activities (N°131/11). Written consent was obtained from all subjects to use anonymous personal and blood data for research purposes. In the informed consensus there was no mention of genetic data.

### Chemicals

Dimethyl disulphide, iodine, 9*cis*-hexadecenoic acid methyl ester and the other commercially available cis and trans FAMEs and CEs were commercially available (Sigma Aldrich, Milan) and used without further purification. Pure samples of 6*cis*-16:1 and 7*cis*-16:1 were also commercially available (Lipidox, Sweden). Chloroform, methanol and *n*-hexane (HPLC grade, Merck) were used without further purification. Non-commercially available trans isomers as standard references were prepared and analyzed as previously described [22, 28–30].

### Blood samples

Blood samples were obtained from a biobank available at the Department of Clinical Medicine and Surgery of the University of Napoli gathered from morbidly obese subjects (n = 50, 16 males and 34 females; age = 35.5 ± 8.9 years, BMI = 41.5 ± 9.0) when they came to the Hospital for routine diagnostics and clinical evaluations. None of them underwent bariatric surgery, whereas all of them adhered to the hospital program for dietary and lifestyle education several months prior to the blood withdrawal. The physio-pathological heterogeneity of the cohort was ascertained, being included patients with diabetes and hypertension comorbidities (12%), hypercholesterolemia (60%) and hypertriglyceridemia (10%). No other subgroups and genotype differentiation was available from the hospital records. The control subjects (n = 50; 10 males and 40 females; age = 42.1 ± 14.0 years) living in the geographical area of the hospital were not diagnosed with any health problems and had BMI values of 22.6 ± 2.4. The dietary habits of patients and controls, coming from the South of Italy (Campania region), were assessed by a food questionnaire and were of a typical Mediterranean diet. Blood was collected early in the morning from the subjects prior to at least 8 hours of fasting. The samples had ethylenediaminetetraacetic acid (EDTA) as the anticoagulant and separation of plasma was effected immediately. The samples of plasma were kept at -70°C until the analysis was performed, and in general were not stored for more than 2 weeks.

### PL isolation and transesterification procedure from erythrocyte membranes

Red blood cells were obtained from fresh EDTA-treated blood samples, separating aged erythrocytes from plasma as previously described [29]; briefly, starting from 1 mL of whole blood, the cell separation was carried out by two consecutive centrifugations, followed by cell washings with phosphate buffer, lysis with distilled water and two subsequent centrifugations, eliminating the aqueous layers, to obtain the erythrocyte membrane pellet. Thin layer chromatography (*n*-hexane/diethyl ether/acetic acid 70/30/1) evidenced the presence of PL and cholesterol as main components. Then, using proper eluent systems and references of all lipid classes [31], the identification of membrane phospholipids, including plasmalogens and sphingomyelin, (SM) was performed. Other components, such as phosphatidic acid (PA), lysophosphatidyl choline (LPC) or lysophosphatidyl ethanolamine (LPE), if present, were not detectable under our conditions. The membrane lipid pellet was extracted by partitioning between aqueous and 2/1 chloroform/MeOH phases. The organic layer was separated and evaporated under vacuum to dryness, then treated at room temperature for 10 min with 0.5 M KOH/MeOH to obtain the transesterification of the fatty acid-containing lipid species into the corresponding fatty acid methyl esters (FAME). It is worth underlining that under these mild conditions only the ester-bound fatty acids can be converted to FAME, whereas ether-bound lipids, such as the two residues present in SM and one residue present in plasmalogens, require different conditions to react. On the basis of the chromatographic and chemical procedures used, we considered that the fatty acids measured in this work mainly are those forming the RBC membrane PL and present as esterified residues. FAME were extracted from the crude alkaline reaction mixture with *n*-hexane and analyzed by gas chromatography as described below. The efficiency of the extraction-transesterification procedures was estimated as 90 ± 3% on the basis of the theoretical yield expected from the starting PL and using an internal calibration standard (C17:0). S1 protocol and S1 Fig give more details of the experimental procedures.

### Extraction of CE from human plasma and formation of FAME

The plasma samples, corresponding to the same blood samples from which RBC membrane PL are extracted, were treated as already reported for the extraction of lipids and separation of the CE fraction [22, 30]. See S1 protocol and S1 Fig for other details of the experimental procedures. If not immediately used in the next step, CE samples were stored in a vial under argon at -20°C. The efficiency of the process was calculated starting from 0.5–0.7 mL of plasma and weighting the isolated CE, confirming previously obtained results [30]. For the transesterification of CE to FAME we checked that an equal recovery yield is obtained using standard references of both CE of 18:0 (saturated) and 18:2 (polyunsaturated) fatty acids.

### GC Analysis of FAME

The analysis of FAME obtained from the RBC membrane PL and plasma CE was performed by dissolution of the weighted sample in 20 μl of *n*-hexane and 1 μl injected directly to the GC under the above detailed analytical conditions. FAME were analyzed by GC (Agilent 6850, Milan), equipped with a 60m × 0.25mm × 0.25μm (50%-cyanopropyl)-methylpolysiloxane column (DB23, Agilent, USA), a flame ionization detector, with injector temperature at 230°C and split injection 50:1. Oven temperature started from 195°C, held for 26 min, followed by an increase of 10°C/min up to 205°C, held for 13 min, followed by a second increase of 30°C/min up to 240°C, and held for 10 min. A constant pressure mode (29 psi) with helium as carrier gas was used. In S2 Protocol other information on calibration procedures and quantitative analysis are given. It is worth mentioning that the same conditions with hydrogen as carrier gas did not give satisfactory separation of C16 MUFA isomers. FAMEs were identified by comparison with the retention times of authentic samples, which are commercially available or synthesized [22, 29, 30]. The list of the examined FAME (corresponding to chromatographic peak areas >97%) in RBC membrane PL and plasma CE are reported in Tables 1 and 2 in μmol/mL ± SEM (standard error of the mean). S1 and S3 Tables report these values in μg/mL ± sd (standard deviation), and S2 and S4 Tables report the corresponding values in %mol (mol/100 mol total fatty acids) ± sd (standard deviation) in both lipid classes, allowing comparison with literature for healthy controls]. S5 Table reports the LOD (limit of detection) and LOQ (limit of quantification) values of the main fatty acid references used in this work.

**Table 1:** Main fatty acid residues of the erythrocyte membrane phospholipids of lean healthy controls and morbidly obese subjects.

Fatty acid methyl ester	Control (n = 50)	Morbidly obese (n = 50)
FAME^1^	(μmol/mL± SEM)^2^	(μmol/mL± SEM)^2^
**16:0**	**2.203±0.011**	2.710±0.015****
**6t- and 9t-16:1**	**nd**	**nd**
**6c-16:1**	**0.019±0.0004**	0.026±0.0004****
**9c-16:1**	**0.041±0.001**	0.078±0.007****
**18:0**	**1.414±0.010**	1.913±0.040****
**9t-18:1**	**0.010±0.0003**	0.017±0.001****
**9c-18:1**	**1.720±0.013**	1.477±0.044****
**11c-18:1**	**0.142±0.001**	0.128±0.003****
**9,12-trans isomers**	**nd**	0.014±0.0003****
**9c,12c-18:2 ω6**	**1.453±0.024**	1.246±0.024****
**8c,11c,14c-20:3 (DGLA) ω6**	**0.231±0.009**	0.268±0.012*
**5c,8c,11c,14c-20:4 ω6**	**1.595±0.003**	1.699±0.044*
**mono trans isomers 20:4**^3^	**0.009±0.0003**	**0.009±0.0009**
**20:5 ω3 (EPA)**	**0.085±0.003**	0.060±0.003****
**22:5 ω3 (DPA)**	**0.273±0.006**	0.128±0.012****
**22:6 ω3 (DHA)**	**0.753±0.015**	0.304±0.015****
**total SFA**	**3.578±0.014**	4.583±0.046****
**total MUFA cis**	**2.011±0.014**	1.784±0.046****
**total MUFA trans**	**0.011±0.0004**	0.018±0.001****
**total PUFA cis**	**4.303±0.037**	3.597±0.056****
**total PUFA trans**	**0.009±0.0003**	0.022±0.0009****
**total TRANS**	**0.020±0.001**	0.040±0.0003****
**total ω6**	**3.246±0.032**	**3.194±0.051**
**total ω3**	**1.133±0.018**	0.499±0.021****

The Table shows the main fatty acid composition of erythrocyte membrane phospholipids expressed as μmol/mL ± SEM as obtained from the gas chromatographic analysis of the corresponding fatty acid methyl esters (FAME), isolated from the blood samples by the procedure described in Materials and methods and S1 Protocol.

^1^ FAME identified by the standard references and quantified as described in Materials and methods and S2 Protocol.

^2^ The values are obtained from the main GC peak areas (>97% of the total peak areas of the chromatogram). Details of the statistical analysis are reported in Materials and methods.

* p value ≤0.05;

**** = p value <0.0001;

nd = not detected.

^3^identified with an appropriate mono-trans lipid library developed as described earlier [28].

**Table 2:** Main fatty acid residues of plasma cholesteryl esters in lean healthy controls and morbidly obese subjects.

Fatty acid methyl ester	Control (n = 50)	Morbidly obese (n = 50)
FAME^1^	(μmol/mL± SEM)^2^	(μmol/mL± SEM)^2^
**16:0**	**1.800±0.044**	1.453±0.037****
**6t-16:1**	**tr**	**tr**
**6c-16:1**	**0.104±0.007**	0.067±0.004****
**9c-16:1**	**0.268±0.019**	0.339±0.026*
**18:0**	**0.157±0.007**	0.188±0.007**
**9t-18:1**	**0.007±0.0003**	0.013±0.0003****
**9c-18:1**	**3.207±0.108**	2.111±0.071****
**11c-18:1**	**0.142±0.007**	0.101±0.003****
**9c,12t-18:2**	**0.007±0.0003**	0.010±0.001**
**9t,12c-18:2**	**0.020±0.003**	0.041±0.007**
**9c,12c-18:2 ω6**	**6.224±0.119**	6.975±0.132****
**8c,11c,14c-20:3 (DGLA) ω6**	**0.128±0.009**	0.184±0.012***
**5c,8c,11c,14c-20:4 ω6**	**0.590±0.016**	1.133±0.031****
**mono trans isomers 20:4**^3^	**0.006±0.0003**	0.019±0.003****
**20:5 ω3 (EPA)**	**0.047±0.003**	**0.044±0.003**
**22:6 ω3 (DHA)**	**0.047±0.006**	0.026±0.003**
**total SFA**	**1.877±0.042**	1.578±0.035****
**total MUFA cis**	**3.869±0.113**	2.708±0.078****
**total MUFA trans**	**0.007±0.0004**	0.014±0.0004****
**total PUFA cis**	**6.571±0.113**	7.840±0.129****
**total PUFA trans**	**0.031±0.003**	0.066±0.006****
**total TRANS**	**0.040±0.003**	0.082±0.007****
**total ω6**	**6.626±0.116**	7.950±0.132****
**total ω3**	**0.094±0.006**	0.070±0.003***

The Table shows the main fatty acid composition of plasma cholesteryl esters expressed as μmol/mL ± SEM as obtained from the gas chromatographic analysis of the corresponding fatty acid methyl esters (FAME), isolated from the plasma by the procedure described in Materials and methods and S1 Protocol.

^1^ FAME identified by the standard references and quantified as described in Materials and methods and S2 Protocol.

^2^ The values are obtained from the main GC peak areas (>97% of the total peak areas of the chromatogram). Details of the statistical analysis are reported in Materials and methods.

* p value ≤0.05;

** p value = 0.01;

*** p value ≤0.001;

**** p value <0.0001;

tr = traces.

^3^identified with an appropriate mono-trans lipid library developed as described earlier [28].

### Dimethyl disulfide adducts for the determination of the double bond position

The treatment of FAME extracts for the preparation of DMDS adducts was performed as previously described [22, 32]. The products were analyzed by GC-MS (Thermo Scientific Trace 1300) equipped with a 15m × 0.25mm × 0.25μm TG-SQC 5% phenyl methyl polysiloxane column, with helium as carrier gas, coupled to a mass selective detector (Thermo Scientific ISQ) with the following oven program: temperature started at 80°C, maintained for 2 min, increased at a rate of 15°C/min up to 140°C, increased at a rate of 5°C/min up to 280°C and held for 10 min.

### Statistical Methods

Statistical analysis was performed using GraphPad Prism 5.0 software (GraphPad Software, Inc., San Diego, CA). We used non-parametric unpaired *t*-test two-tailed with 95% confidence interval. Results in Tables 1 and 2 are reported as mean values ± standard error of the mean (SEM). The statistical significance is calculated based on the SEM. S1–S4 Tables report mean values ± standard deviation (SD).

## Results

### Fatty acid analysis and individuation of C16 MUFA isomers

The groups of morbidly obese patients and lean healthy controls were equal in number (n = 50). The former (16 males and 34 females; age = 35.5 ± 8.9 years, BMI = 41.5 ± 9.0) were present for routine diagnostics and clinical evaluations, following a hospital program for dietary and lifestyle control, whereas the lean subjects (10 males and 40 females; age = 42.1 ± 14.0 years; BMI = 22.6 ± 2.4) living in the same region did not have signs of health problems and had closely matching ages. The morbidly obese cohort was heterogeneous as far as physio-pathological conditions are concerned, including patients with diabetes or hypertension comorbidities (12%), hypercholesterolemia (60%) and hypertriglyceridemia (10%). Within this patient population, we could not discriminate genotype or other subgroups, with the samples being obtained from a biobank made of routine check-up analyses. This work is based on the fatty acid differences between morbidly obese and lean healthy subjects, not referring at this stage to a specific obesity phenotype. After the fatty acid compositions are obtained from the control group, the results were also checked against the typical values reported in human healthy cohorts, indeed verifying our control values are in agreement with those expected [7]. We focused our work on the comparative fatty acid analysis of RBC membrane PL and plasma CE fatty acids, with particular regard to the satisfactory separation and identification of the C16 MUFA positional isomers and metabolites of delta-6 and delta-9 desaturase enzymes (shown in Fig 1), for a correct interpretation of the desaturase pathways. The parallel evaluation of the C16 MUFA family, in two lipid classes of the same individual, is useful for the full understanding of lipidomic profiles and evaluation of differences in health and disease conditions. The FAME identification and quantitation by GC were performed under the optimal conditions for the separation of delta-6 and delta-9 positional isomers, as well as of their geometrical trans isomers [22] (see also Supporting Information). We further demonstrated that the carrier gas is important to have satisfactory separation, being a net advantage the use of helium instead of hydrogen with a column length of at least 60 m. Since these conditions cannot resolve 7*cis*-16:1 that coelutes with 6*cis*-16:1 isomers, a derivatization step was introduced in the protocol to unequivocally assign the fatty acid double bond position, using dimethyl disulfide (DMDS). This reagent adds to the double bond giving the corresponding DMDS-fatty acid adducts [32], that are examined by mass spectrometry giving diagnostic fragmentation patterns for the location of the double bond along the fatty acyl chain, fully solving the C16 MUFA isomer assignment [22]. In our samples we established unambiguously that the 7*cis*-16:1 isomer is below the detection limits of our method. The fatty acid compositions of RBC membrane PL and plasma CE in the two subject groups are reported in Tables 1 and 2 as μmol/mL with the standard deviation expressed as SEM (standard error of the mean). It is worth underlining that the lipid extracts were checked by appropriate TLC analysis for the lipid classes [31], as described in Materials and methods, and that alkaline conditions of transesterification employed here can transform only the ester-bound,lipids, and not ether-bound lipids or free fatty acids, to FAME. The FAME values are also provided as μg/mL ± standard deviation (SD) (S1 and S3 Tables) as well as molar percentages (% mol) ± standard deviation (SD), the latter considering 100 moles of the detected fatty acids, which in their turn correspond to >97% of the GC peak areas (S2 and S4 Tables). The %mol unit is useful to compare our results with previously reported data for erythrocyte membrane PL and plasma CE fatty acids, as result of a meta analysis [7].

**Fig 2:**
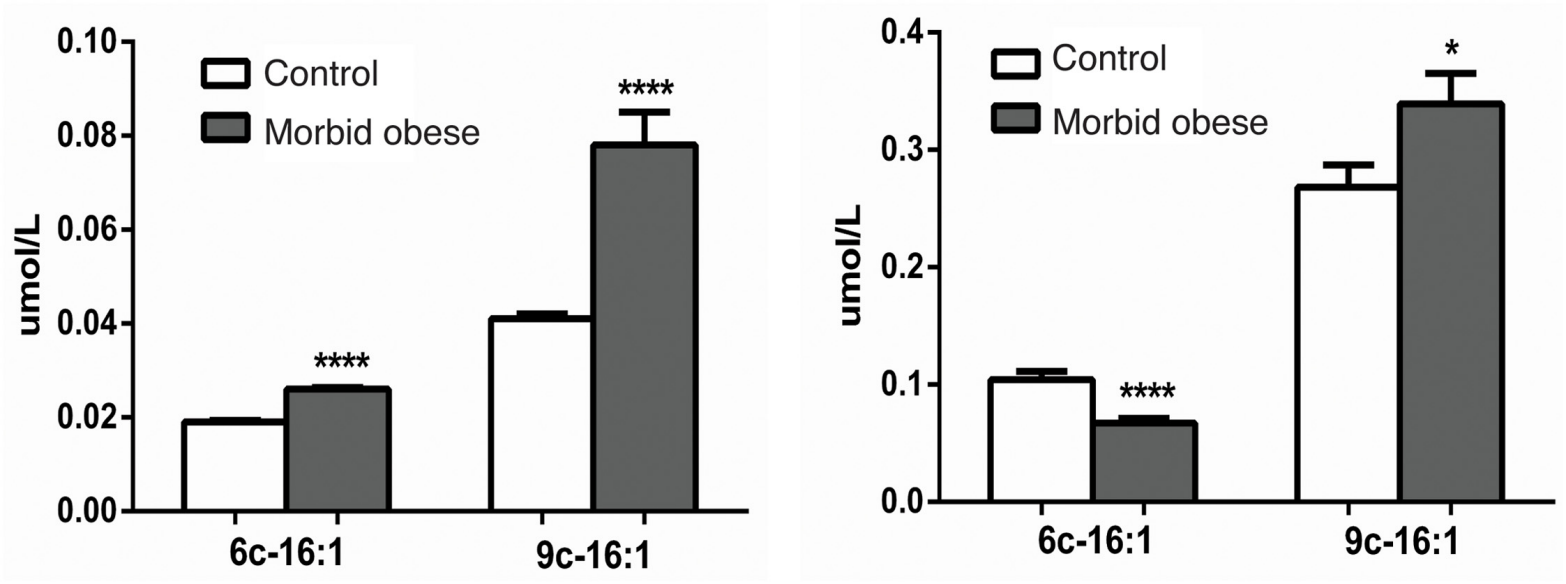
Comparison of the sapienic and palmitoleic acid contents (μmol/mL) in RBC membrane PL (A) and plasma CE (B). Significant increases of palmitoleic and sapienic acids are found in PL of morbidly obese subjects compared to controls (both with p < 0.0001), whereas sapienic acid is diminished in CE of morbidly obese subjects (with p < 0.0001), with an opposite trend compared to palmitoleic acid (p ≤ 0.05). Values and error bars (SEM) are reported in Tables 1 and 2.

While comparison of plasma fractions with adipose tissue lipids or only RBC membrane lipids have been previously described [7, 11–15, 25, 26], to our knowledge this is the first time that these two lipid species are compared in human morbid obesity. Palmitoleic acid levels were increased significantly in morbidly obese patients both in RBC membrane PL and plasma CE (p values ≤ 0.0001 and ≤ 0.05, respectively, see Tables 1 and 2). The new aspect in this work is the evaluation of sapienic acid; as shown in Fig 2, this fatty acid is significantly increased in the RBC membrane PL of morbid obese subjects (p value < 0.0001) as found for palmitoleic acid (Fig 2A), suggesting that the metabolic conversion of palmitic acid by delta-9 desaturase (with formation of palmitoleic acid) is accompanied by an enhanced, though unusual, delta-6 desaturase pathway using the same substrate—palmitic acid. Evaluating plasma CE in morbidly obese subjects, palmitoleic acid remained with an increase compared to lean healthy subjects, whereas sapienic acid was significantly diminished (p value < 0.0001) (Fig 2B).

Other interesting fatty acid changes could be observed when the two lipid classes are compared. The trends and statistical significances of some of them are summarized in Table 3. In particular, increases of stearic acid (C18:0) and omega-6 C20 PUFA, i.e., 8*cis*,11*cis*,14*cis*-20:3 (dihomo-gamma-linolenic acid, DGLA) and 5*cis*,8*cis*,11*cis*,14*cis*-20:4 (arachidonic acid), were found in both lipid classes, as well as the decrease of oleic (9*cis*-18:1) and DHA (C22:6 omega-3). Palmitic acid and linoleic acid (9*cis*,12*cis*-18:2, omega-6) gave opposite trends in plasma CE and membrane PL.

**Table 3:** Some of the fatty acid trends to increase (↑) or decrease (↓) for morbidly obese subjects, as compared with lean healthy individuals, in RBC membrane PL and plasma CE.

Fatty acid	RBC PL^1^	PLASMA CE^1^
**16:0**	↑****	↓****
**18:0**	↑****	↑**
**6c-16:1**	↑****	↓****
**9c-16:1**	↑****	↑*
**9c-18:1**	↓****	↓****
**9c,12c-18:2 ω6**	↓****	↑****
**8c,11c,14c-20:3 (DGLA)**	↑*	↑***
**5c,8c,11c,14c-20:4 ω6**	↑*	↑****
**22:6 ω3 (DHA)**	↓****	↓**

Table 3 shows at a glance the significant fatty acid trends in morbidly obese subjects using the values of Tables 1 and 2 in comparison with lean healthy subjects, underlining the behavior of palmitic and sapienic acids, that increase in RBC membrane PL and decrease in plasma CE, in contrast with that of linoleic acid, decreasing in RBC membrane PL and increasing in plasma CE.

^1^Details of the statistical analysis are reported in Materials and methods.

* p value ≤0.05;

** p value = 0.01;

*** p value ≤0.001;

**** p value <0.0001.

### Evaluation of desaturase indexes

The desaturase indexes calculated in both RBC membrane PL and plasma CE of morbidly obese and lean healthy controls are shown in Table 4. They were obtained by using the ratio of the quantities in %mol of the product and corresponding precursor fatty acid reported in S2 and S4 Tables. The ratio of 9*cis*-16:1 and 16:0 gives the delta-9 desaturase index on C16 fatty acids (SCD-16 index). In both RBC membrane PL and plasma CE the SCD-16 index gave statistical significance, with slightly different p values (0.01 vs. ≤0.05, respectively).

**Table 4:** Enzymatic indexes obtained from the quantities of the corresponding fatty acids in molar percentages (%mol) presented in S2 and S4 Tables.

Enzymatic index	Control	Morbidly obese	Control	Morbidly obese
	RBC PL	RBC PL^1^	PLASMA CE	PLASMA CE^1^
	(n = 50)	(n = 50)	(n = 50)	(n = 50)
**6c-16:1/16:0 (D6D)**^#^	0.009±0.002	0.010±0.003	0.058±0.048	0.046±0.037
**9c-16:1/16:0 (SCD-16)**^†^	0.018±0.005	0.029±0.028**	0.149±0.131	0.234±0.201*
**8c,11c,14c-20:3/9c,12c-18:2 (D6D + ELO)**^§^	0.159±0.071	0.215±0.129**	0.021±0.016	0.026±0.020
**5c,8c,11c,14c-20:4/8c,11c,14c-20:3 (D5D)**^≠^	6.918±2.932	6.331±4.202	4.620±3.907	6.152±5.012

Table 4 reports the values ± SD of desaturase indexes (delta-6 desaturase, D6D; delta-9 desaturase, SCD-16; delta-5 desaturase, D5D) and one combined desaturase and elongase index (D6D + ELO, elongase) obtained from the corresponding substrate and metabolite fatty acids in the RBC membrane PL and plasma CE.

^#^D6D, Delta-6 desaturase index;

^†^ delta-9 desaturase index (SCD-16);

^§^this index is referred to the metabolic transformation by delta-6 desaturase and elongase (ELO) enzymes;

^≠^D5D, delta-5 desaturase index.

^1^Details of the statistical analysis are reported in Materials and methods. The statistical significance is estimated based on Standard Error of the Mean values (SEM, see Tables 1 and 2).

* p value ≤0.05;

** p value = 0.01.

Delta-6 desaturase (D6D) generally is the first enzyme used in the essential PUFA conversions (12–14), and in our work the index was calculated for the first time in case of the C16MUFA pathway from palmitic acid to sapienic acid (see Fig 1). The D6D index shows opposite but not significant trends for this fatty acid in RBC PL and plasma CE (compare Tables 3 and 4). The D6D index was estimated in the PUFA omega-6 pathway and in combination with the elongase step, going from linoleic acid to DGLA. This index was found significant only in RBC membrane PL with an increased value in morbidly obese patients (p = 0.01). The delta-5 desaturase index (D5D), evaluated in the PUFA omega-6 fatty acids using the DGLA transformation to arachidonic acid gave no significance.

## Discussion

Palmitic acid transformations and desaturase indexes are attracting considerable interest for lipidomic studies, looking for biomarkers of the *de novo* lipogenesis pathways associated to metabolic diseases [33–36]. Our findings on morbidly obese patients cannot be extrapolated to the obesity phenotype neither can be referred to patient subgroups or genotype. The present work has a methodological rather than a pure diagnostic value, pointing out that: i) the correct evaluation of the fatty acid structures is needed for biomarker discovery, and ii) the comparison of two well metabolically connected lipid classes, such as erythrocyte membranes PL and plasma CE, is needed to formulate and interpret lipidomic profiles. For this purpose, on the basis of the chemical and analytical procedures used in the present work and described in Materials and methods, we isolated the RBC membrane pellet, ascertaining the presence of phospholipids (PC, PE, PI, PS) and subsequently obtaining the fatty acid residues by alkaline hydrolysis conditions as the corresponding FAME. The presence of other lipid classes cannot be ruled out, although they were not detected under our experimental conditions. Plasma CEs were separated from the same blood sample and their FAME composition was also determined to compare with the RBC membrane PL, eliminating the influence of diet in each subject cohort. Here we report for the first time the presence of sapienic acid in erythrocyte membrane PL and its significant increase comparing morbidly obese subjects with lean healthy controls. This is the metabolite of palmitic acid by delta-6 desaturase activity previously reported by us in circulating lipids, such as lipoproteins and plasma CE [22]. Others reported it as “negative control” for the evaluation of plasma SCD index, i.e., the percentage of palmitic acid transformation not following the SCD-16 pathway [16] (see Fig 1), underlining that mass spectrometry tools such as neutral loss strategy, are unsuccessful with positional isomers [37]. The determination of positional isomers is important for the correct individuation of the MUFA pathway and calculation of desaturase index as emerging biomarker in health and disease conditions. Research on the identification and quantitation of the lipid double bond location shows a renovated interest, as demonstrated by a recent paper that proposed shotgun lipidomics of lipid extracts previously treated by the Paternò-Buchi reaction as derivatization method to discriminate positional isomers in different classes of lipids [38]. In our protocol the derivatization of FAMEs as DMDS adducts and evaluation of these DMDS-adducts, together with the initial gas chromatographic separation of FAME isomers, furnish a double-checked recognition protocol for the assignment of the double bond position of the C16 MUFA isomers [22, 32]. Another important issue emerged from our data, regarding the lipid compartment used for the estimation of lipidomics pathways. Indeed, attention has been raised on serum phospholipids, that are not reliable due to the significant exchange of fatty acids with systemic circulation between lipid pools [39], whereas the SCD index estimated from CE mainly reflects the liver enzymatic activities [13]. In our case, the parallel monitoring of plasma CE and RBC membrane PL highlighted that some fatty acids follow the same trends, such as stearic, palmitoleic, oleic, DGLA and arachidonic acids, whereas palmitic and sapienic acids are increased in RBC membrane PL and decreased in plasma CE, and linoleic acid follows the opposite trend. This result encourages the extension of such comparative evaluation to large cohorts, also in connection with the LCAT functioning already found relevant for cardiovascular diseases and atherosclerosis [26, 40–42]. The increase of palmitic acid, which is known to be associated positively with adiposity [33], is appreciable only in RBC membrane PL, whereas linoleic acid increase is seen only in plasma CE. The latter result can indicate a different activity of LCAT or of other enzymes involved in CE biosynthesis, as well as of the kinetics of incorporation of fatty acids into lipid classes, which is known for healthy subjects upon supplementation [43]. From our results sapienic acid emerges as an interesting metabolite to be followed up in physio-pathological conditions, also in connection with the emerging importance of genetic variations of lipid desaturase [44]. Finally, using synthetically available molecular libraries of trans fatty acids [24], significant increases of the geometrical trans fatty acid isomers were found that confirm previously reported results [25, 26]. In the work described herein, trans fatty acids were detected in both MUFA and PUFA families, with higher values in plasma CE than in RBC membrane PL and statistical significance (compare Tables 1 and 2; p values ≤ 0.0001). Further work is needed to assess the relationship between the exposure of unsaturated fats circulating in the plasma to free radicals and the extent of their geometrical double bond conversion, in comparison with the same process occurring to membrane unsaturated lipids. Lipid isomerization can acquire more significance in metabolic diseases, considering the adipogenic effect of trans compared to cis monounsaturated fatty acid isomers [45].

Overall, our results highlight the importance of the hexadecenoic fatty acid family, that goes beyond the significance of each lipid class, with the exact identification of positional isomers that comes first, in view of a better understanding of the desaturase enzymatic activities and of a correct assignment of lipidomic pathways.

## Supporting Information

S1 File Contains details of the blood sample treatment for separation of the lipids and transesterification, gas chromatographic calibration and quantitation, as well as S1–S4 Tables as mentioned in the main text. (PDF)

S1 Fig The workflow of the experimental procedures for blood lipid separation and fatty acid methyl ester (FAME) formation used for GC analysis. (TIF)
