# Supplementary material for: Hexadecenoic Fatty Acid Isomers in Human Blood Lipids and Their Relevance for the Interpretation of Lipidomic Profiles
Source: PLoS One. 2016 Apr 5;11(4):e0152378. doi: 10.1371/journal.pone.0152378 (PMC4821613; doi:10.1371/journal.pone.0152378)
Supplement: S1 File — (PDF) [file pone.0152378.s001.pdf]

Hexadecenoic fatty acid isomers in human blood lipids and their relevance for the interpretation of lipidomic profiles

Anna Sansone[1], Evanthia Tolika[1], Maria Louka[2], Valentina Sunda[2], Simone Deplano[2], Michele Melchiorre[2], Dimitrios Anagnostopoulos[3], Chryssostomos Chatgilialoglu[3], Cesare Formisano[4], Rosa Di Micco[4], Maria Rosaria Faraone Mennella[5], Carla Ferreri[*,1]

Corresponding author: Carla Ferreri; email: carla.ferreri@isof.cnr.it

S1 Protocol. Details of the blood sample treatment for separation of the lipids and transesterification.

The EDTA-treated blood samples (volume 1 mL) were used either for the plasma cholesteryl ester (CE) and the RBC membrane phospholipid (PL) isolation. S1 Fig in S1 Text describes the workflow of the separation of the two lipid classes and work-up for the fatty acid methyl ester analysis.

The yields of fatty acid methyl ester transformation from membrane phospholipids and cholesteryl esters fractions were also evaluated by performing the procedures with lipid-containing C18:0 (stearic acid methyl ester) and C18:2 (linoleic acid methyl ester) as standard references. We estimated that lipids were satisfactorily extracted and transesterified from the samples (total recovery > 90%).

As reported previously (2), the transesterification of cholesteryl esters to FAME was carried out by mild conditions at room temperature, under argon and dark. We recommend checking the conditions of transesterification of biological samples, by testing not only saturated fatty acids but also C18:2 components as compounds for the calculation of the reaction yield.

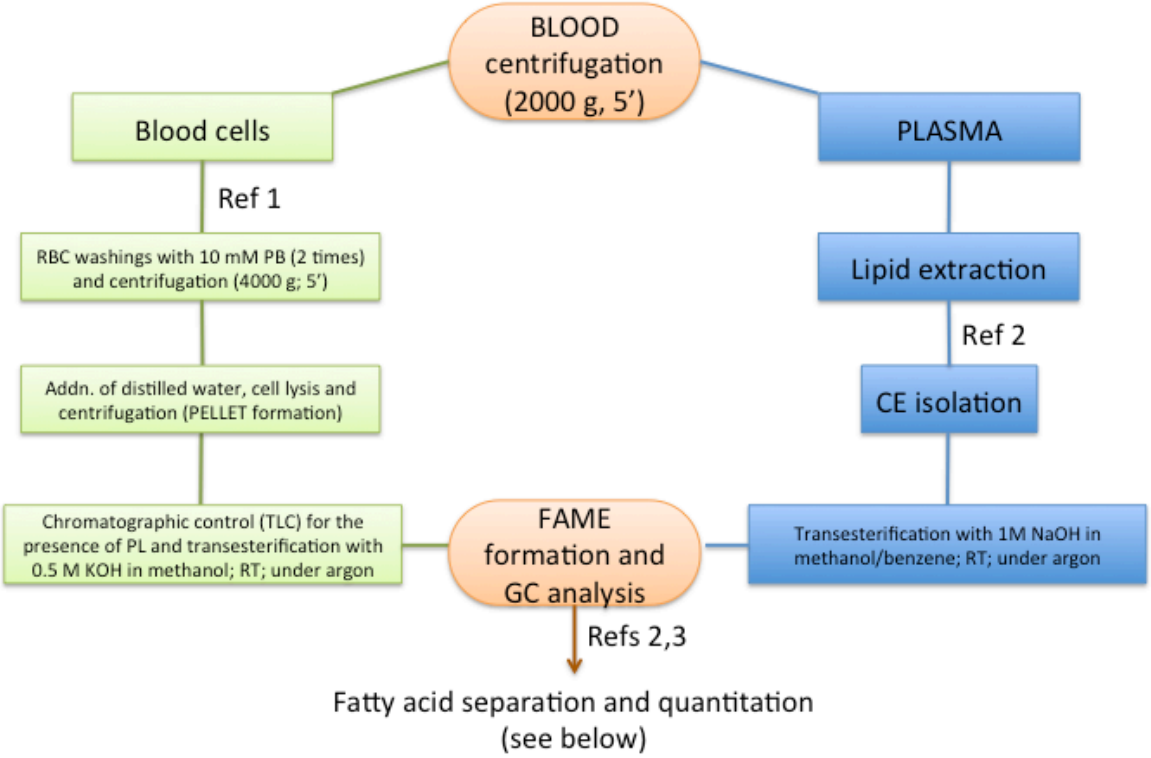

S1 Fig. The workflow of the experimental procedures for blood lipid separation and fatty acid methyl ester (FAME) formation used for GC analysis. After collection and centrifugation of the blood (1 mL), plasma and blood cells are separated and then treated separately for the lipid extraction and transformation of ester-bound fatty acids into the corresponding FAME for GC analysis.

S1 Table. Main fatty acid residues (µg/mL± SD) of the erythrocyte membrane phospholipids of controls and morbidly obese subjects.

Fatty acid methyl ester FAME[1]	Control (n=50) (µg/mL± SD)[2]	Morbidly obese (n=50) (µg/mL± SD)[2]
**16:0**	0.596±0.019	0.733±0.030****
**6t- and 9t-16:1**	nd	nd
**6c-16:1**	0.005±0.001	0.007±0.001****
**9c-16:1**	0.011±0.002	0.021±0.017****
**18:0**	0.422±0.022	0.571±0.084****
**9t-18:1**	0.003±0.001	0.005±0.002****
**9c-18:1**	0.510±0.027	0.438±0.091****
**11c-18:1**	0.042±0.003	0.038±0.007***
**Mono trans isomers 18:2**	nd	0.004±0.001****
**9c,12c-18:2 ω6**	0.428±0.050	0.367±0.046****
**8c,11c,14c-20:3 (DGLA) ω6**	0.074±0.020	0.086±0.031*
**5c,8c,11c,14c-20:4 ω6**	0.508±0.047	0.541±0.102*
**mono trans isomers 20:4**[3]	0.003±0.001	0.003±0.002
**20:5 ω3 (EPA)**	0.027±0.008	0.019±0.009****
**22:5 ω3 (DPA)**	0.094±0.011	0.044±0.028****
**22:6 ω3 (DHA)**	0.258±0.037	0.104±0.034****
**total SFA**	1.018±0.029	1.304±0.090****
**total MUFA cis**	0.568±0.027	0.504±0.092****
**total MUFA trans**	0.003±0.001	0.005±0.002****
**total PUFA cis**	1.389±0.082	1.161±0.126****
**total PUFA trans**	0.003±0.001	0.007±0.002****
**total TRANS**	0.006±0.002	0.012±0.001****
**total ω6**	1.010±0.071	0.994±0.116
**total ω3**	0.379±0.039	0.167±0.050****

The main fatty acid composition of erythrocyte membrane phospholipids is reported as µg/mL± SD, corresponding to the data in Table 1 of the main text reported as µmol/mL± SEM.

[1] FAME identified by the standard references and quantified as described in Materials and methods.
[2] The values are obtained from the main GC peak areas (>97% of the total peak areas of the chromatogram). Details of the statistical analysis are reported in Materials and methods. The statistical significance is estimated based on Standard Error of the Mean (SEM, see Table 1 in the Main text): * p value ≤0.05; ****= p value <0.0001; nd = not detected.

[3]identified with an appropriate mono-trans lipid library developed as described earlier (1).

S2 Table. Main fatty acid residues (% mol ± SD) of the erythrocyte membrane phospholipids of control and morbidly obese subjects.

Fatty acid methyl esters FAME[1]	Control (n=50) (%mol± SD)[2]	Morbidly obese (n=50) (%mol± SD)[2]
**16:0**	22.17±1.36	26.86±2.66****
**6t – and 9t-16:1**	nd	nd
**6c-16:1**	0.19±0.04	0.26±0.05****
**9c-16:1**	0.41±0.09	0.78±0.67***
**18:0**	14.22±1.16	18.96±3.89****
**9t-18:1**	0.10±0.04	0.17±0.08****
**9c-18:1**	17.31±1.43	14.65±3.89****
**11c-18:1**	1.43±0.14	1.27±0.31**
**mono trans isomers 18:2**	nd	0.23±0.04****
**9c,12c-18:2 ω6**	14.62±2.14	12.35±2.27****
**8c,11c,14c-20:3 (DGLA) ω6**	2.32±0.70	2.66±1.11
**5c,8c,11c,14c-20:4 ω6**	16.05±1.96	16.84±4.15
**mono trans isomers 20:4**	0.10±0.04	0.10±0.05
**20:5 ω3 (EPA)**	0.86±0.28	0.60±0.32****
**22:5 ω3 (DPA)**	2.75±0.40	1.27±0.88****
**22:6 ω3 (DHA)**	7.58±1.31	3.01±1.16****
**total SFA**	36.39±2.10	45.82±5.66****
**total MUFA cis**	19.34±1.50	16.96±4.10***
**total MUFA trans**	0.10±0.04	0.17±0.08****
**total cis PUFA**	44.18±3.93	36.73±6.02****
**total trans PUFA**	0.10±0.03	0.33±0.10****
**total TRANS**	0.20±0.04	0.50±0.13****
**total ω6**	32.99±3.33	31.85±5.51
**total ω3**	11.19±1.49	4.88±1.59****

The main fatty acid composition of erythrocyte membrane phospholipids is reported as %mol ± SD, corresponding to the data in S1 Table reported as μg/mL± SD. The fatty acid composition of the healthy controls in %mol values is in agreement with that reported from meta analysis of the healthy population (4).

[1] FAME identified by the standard references as described in Materials and methods.

[2] The values are obtained from the main GC peak areas (>97% of the total peak areas of the chromatogram). Details of the statistical analysis are reported in Materials and Methods. The statistical significance is estimated based on Standard Error of the Mean (SEM, see Table 1 in the Main text): ** p value = 0.01; *** p value ≤ 0.001;**** p value < 0.0001; nd = not detected.

S3 Table. Main fatty acid residues (μg/mL± SD) of plasma cholesteryl esters in controls and morbidly obese subjects.

Fatty acid methyl ester (FAME)[1]	Control (n=50) (μg/mL± SD)[2]	Morbidly Obese (n=50) (μg/mL± SD)[2]
**16:0**	0.487±0.085	0.393±0.068****
**6t-16:1**	tr	tr
**6c-16:1**	0.028±0.013	0.018±0.008****
**9c-16:1**	0.072±0.037	0.091±0.047*
**18:0**	0.047±0.012	0.056±0.015**
**9t-18:1**	0.002±0.001	0.004±0.001****
**9c-18:1**	0.951±0.225	0.626±0.148****
**11c-18:1**	0.042±0.012	0.030±0.009****
**9c,12t-18:2**	0.002±0.001	0.003±0.002**
**9t,12c-18:2**	0.006±0.005	0.012±0.011***
**9c,12c-18:2 ω6**	1.833±0.249	2.054±0.279****
**8c,11c,14c-20:3 (DGLA) ω6**	0.041±0.019	0.059±0.026***
**5c,8c,11c,14c-20:4 ω6**	0.188±0.036	0.361±0.070****
**mono trans isomers 20:4[3]**	0.002±0.001	0.006±0.004****
**20:5 ω3 (EPA)**	0.015±0.007	0.014±0.006
**22:6 ω3 (DHA)**	0.016±0.012	0.009±0.006***
**total SFA**	0.534±0.085	0.449±0.069****
**total MUFA cis**	1.093±0.228	0.765±0.155****
**total MUFA trans**	0.002±0.001	0.004±0.001****
**total PUFA cis**	2.093±0.252	2.497±0.288****
**total PUFA trans**	0.010±0.005	0.021±0.011****
**total TRANS**	0.012±0.005	0.025±0.012****
**total ω6**	2.062±0.252	2.474±0.288****
**total ω3**	0.031±0.013	0.023±0.008***

The main fatty acid composition of plasma cholesteryl esters is reported as μg/mL± SD, corresponding to the data in Table 2 of the main text reported as μmol/mL± SEM.

[1] FAME identified by the standard references as described in Materials and methods.

[2]The values are obtained from the main GC peak areas (>97% of the total peak areas of the chromatogram). It is possible to convert these values in μmol/L by applying the following calculation:([μg/mL value/molecular weight]/1000). For example: palmitic acid in erythrocyte membrane PL = 0.487 μg/mL corresponds to 0.00000180 μmol/L. Details of the statistical analysis are reported in Materials and methods. The statistical

significance is estimated based on Standard Error of the Mean (SEM, see Table 1 in the Main text): ** p value = 0.01; *** p value ≤0.001;**** p value <0.0001; tr= traces.
[3]identified with an appropriate mono-trans lipid library developed as described earlier (2).

S4 Table. Main fatty acid residues (% mol ± SD) of plasma cholesteryl esters of controls and morbidly obese subjects.

Fatty acid methyl esters FAME[1]	Control (n=50) (%mol± SD)[2]	Morbidly Obese (n=50) (%mol± SD)[2]
**16:0**	14.10±3.78	11.43±3.00***
**6t-16:1**	tr	tr
**6c-16:1**	0.82±0.46	0.53±0.28***
**9c-16:1**	2.10±1.28	2.67±1.62*
**18:0**	1.23±0.43	1.48±0.53*
**9t-18:1**	0.05±0.03	0.11±0.04****
**9c-18:1**	25.12±8.30	16.61±5.42****
**11c-18:1**	1.11±0.42	0.80±0.31****
**9c,12t-18:2**	0.05±0.03	0.08±0.06**
**9t,12c-18:2**	0.16±0.15	0.32±0.32**
**9c,12c-18:2 ω6**	48.75±11.19	54.88±12.38*
**8c,11c,14c-20:3 (DGLA) ω6**	1.00±0.56	1.45±0.77**
**5c,8c,11c,14c-20:4 ω6**	4.62±1.32	8.92±2.53****
**mono trans isomers 20:4**	0.05±0.03	0.15±0.11****
**20:5 ω3 (EPA)**	0.37±0.21	0.35±0.18
**22:6 ω3 (DHA)**	0.37±0.31	0.21±0.16**
**total SFA**	15.33±3.92	12.91±3.18**
**total MUFA cis**	29.15±8.79	20.61±6.03****
**total MUFA trans**	0.05±0.03	0.11±0.05****
**total PUFA cis**	55.11±11.87	65.81±13.59****
**total PUFA trans**	0.26±0.16	0.55±0.36****
**total TRANS**	0.31±0.16	0.66±0.38****
**total ω6**	54.37±11.79	65.25±13.54****
**total ω3**	0.74±0.39	0.56±0.25**

The main fatty acid composition of plasma cholesteryl esters is reported as %mol ± SD, corresponding to the data in S3 Table reported as µg/mL± SD. The fatty acid composition of the healthy controls in %mol values is in agreement with that reported from meta analysis of the healthy population (4).

[1] FAME identified by the standard references as described in Materials and methods.

[2]The values are obtained from the main GC peak areas (>97% of the total peak areas of the chromatogram). Details of the statistical analysis are reported in Materials and methods. The statistical significance is estimated based on Standard Error of the Mean (SEM, see Table 1 in the Main text): * p value ≤0.05; ** p value = 0.01; *** p value ≤0.001;**** p value <0.0001; tr = traces.

S2 Protocol. Details of gas chromatographic calibration procedures and quantitative analysis

The internal standard of C17:0 was added in the FAME samples at known concentration. 1 μL of the FAME mixture in *n*-hexane solution was injected in the GC equipment using the split mode (50:1) with the conditions reported in the text. The results of FAME of RBC membrane PL and plasma CE in controls and morbid obese subjects are reported in μg/mL ± SEM (Standard Error of the Mean) in Tables 1 and 2 of the main text. The Tables in S1 Text report the results as μg/mL ± SD (standard deviation) and as %mol ± SD of the two lipid classes.

LOD (Limit of detection) and LOQ (Limit of quantification) values of the GC instrument used in this work were calculated using standard references for saturated (SFA), monounsaturated (MUFA) and polyunsaturated (PUFA) fatty acids, prepared from a 2 mg/mL solution of each standard and diluted to a series of appropriate concentrations with *n*-hexane.

Other details of the calibration method and repeatability were previously described (3).

S5 Table. LOD (Limit of detection) and LOQ (Limit of quantification) values of the relevant SFA, MUFA and PUFA standard references.

FAME	LOD (μg/mL)	LOQ (μg/mL)	Correlation factor (R^2)
16:0	0.0005	0.0012	0.9979
9c-16:1	0.0012	0.0030	0.9950
6c-16:1	0.0010	0.0020	0.9976
9c- 18:1	0.0012	0.0020	0.9965

**18:2 w6**	**0.0012**	**0.0022**	**0.9988**
**20:4 w6**	**0.0012**	**0.0020**	**0.9951**

References

1. Ghezzo A, Visconti P, Abruzzo PM, Bolotta A, Ferreri C, Gobbi G, et al. Oxidative Stress and Erythrocyte Membrane Alterations in Children with Autism: Correlation with Clinical Features. PLoS One. 2013;8(6):e66418.
2. Melchiorre M, Torreggiani A, Chatgilialoglu C, Ferreri C. Lipid markers of "geometrical" radical stress: synthesis of monotrans cholesteryl ester isomers and detection in human plasma. J Am Chem Soc. 2011;133(38):15184-90.
3. Sansone A, Melchiorre M, Chatgilialoglu C, Ferreri C. Hexadecenoic fatty acid isomers: a chemical biology approach for human plasma biomarker development. Chem Res Toxicol. 2013;26(11):1703-9.
4. Hodson L, Skeaff CM, Fielding BA. Fatty acid composition of adipose tissue and blood in humans and its use as a biomarker of dietary intake. Prog Lipid Res. 2008;47(5):348-80.
