# Supplementary figures and images for: Hexadecenoic Fatty Acid Isomers in Human Blood Lipids and Their Relevance for the Interpretation of Lipidomic Profiles
Source: PLoS One. 2016 Apr 5;11(4):e0152378. doi: 10.1371/journal.pone.0152378 (PMC4821613; doi:10.1371/journal.pone.0152378)

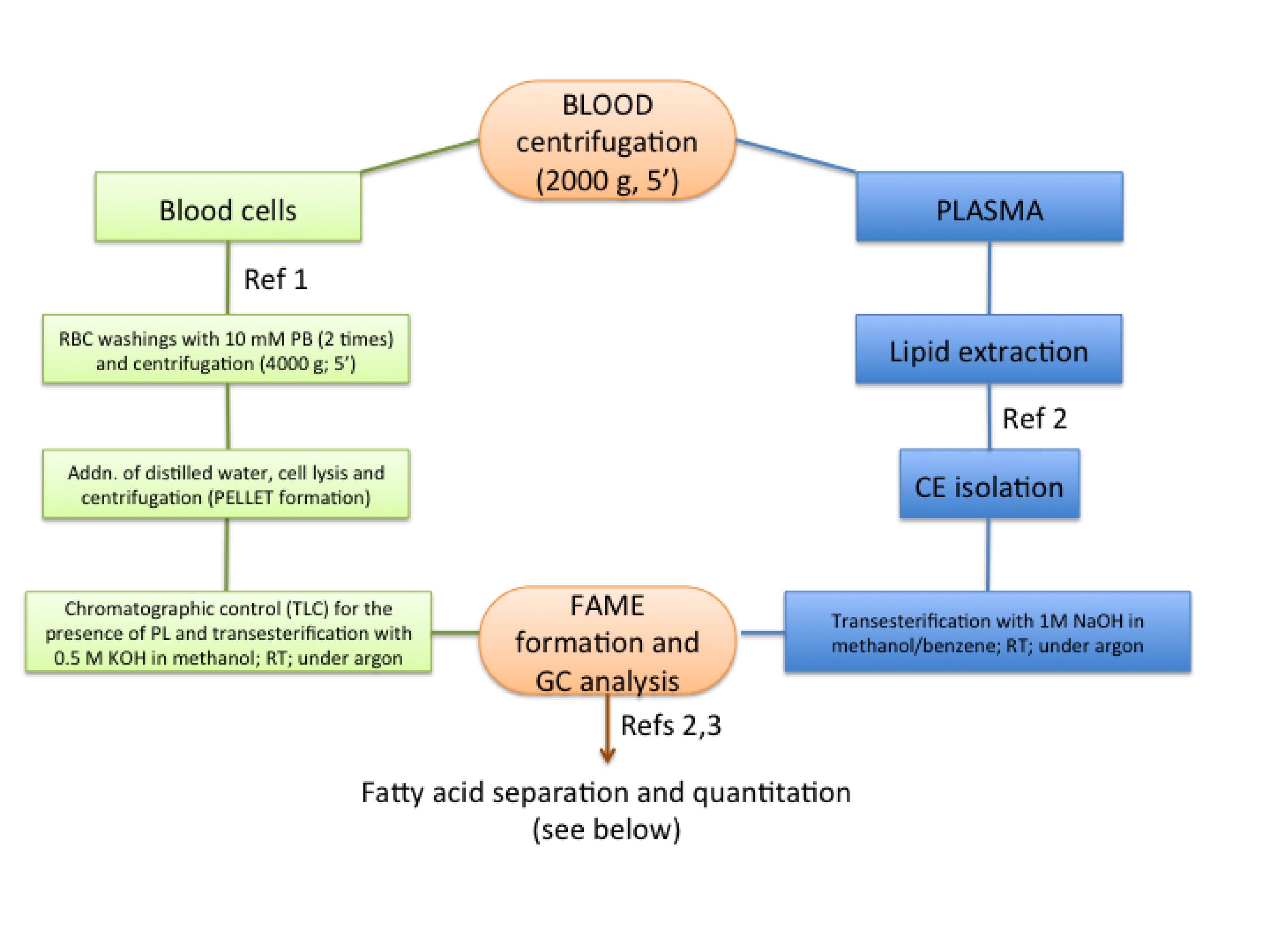

Supplement: S1 Fig — (TIF) [file pone.0152378.s002.tif]
